# High-yield production of lignin nanoparticle photonic glasses†

**DOI:** 10.1039/d4gc05797j

**Published:** 2025-01-17

**Authors:** Unnimaya Thalakkale Veettil, Alberto J. Huertas-Alonso, Tomás S. Plivelic, Mika H. Sipponen

**Affiliations:** a Department of Materials and Environmental Chemistry, Stockholm University SE-106 91 Stockholm Sweden mika.sipponen@mmk.su.se albertojose.huertasalonso@mmk.su.se; b MAX IV Laboratory, Lund University Lund Sweden; c Wallenberg Wood Science Center, Department of Materials and Environmental Chemistry, Stockholm University SE-10691 Stockholm Sweden

## Abstract

Lignin has emerged as a sustainable alternative to fossil-based polymers in advanced materials such as photonics. However, current methods for preparing photonic lignin materials are limited by non-benign organic solvents and low production yields. In this work, we present a highly efficient process that enables the production of photonic glasses with yields ranging from 48% to 72%, depending on the size of the lignin nanoparticles obtained from herbaceous soda lignin, softwood kraft lignin, and hardwood organosolv lignin. The hydrodynamic diameter of lignin nanoparticles can be regulated by the rate of water addition to the lignin–ethanol solution. We demonstrate that this control over particle size allows for tailoring the color of the photonic glass across the visible spectrum.

## Abstract 2

High-yield lignin nanoparticles produced using ethanol as solvent generate photonic glasses with tunable colors, offering a sustainable route to advanced materials from diverse lignin sources.

## Graphical abstract

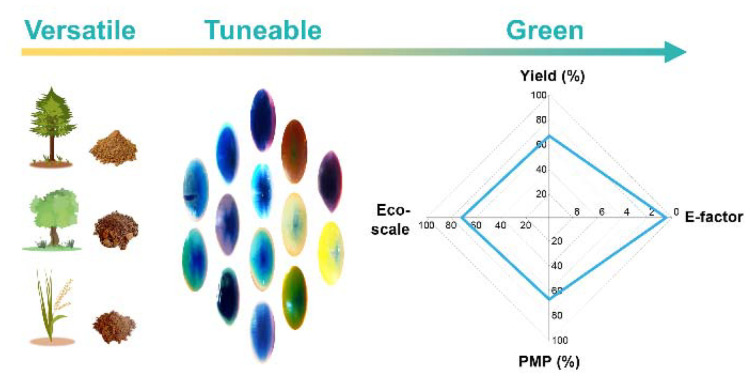

**Green foundation**

1. We obtained structural colors from lignin nanoparticles *via* a simple centrifugation process, despite their broad size distribution. The method is versatile across lignin sources: softwood, hardwood, and grasses.

2. We successfully produced structural colors with a high feedstock conversion efficiency of up to 72% using ethanol as a green solvent. Mass yield, Eco-scale, E-factor, and Process Mass Productivity were used to quantitatively evaluate these features.

3. The structural colors should be made resistant to drying and mechanically robust to prevent cracking. Additionally, developing alternative methods to assemble lignin nanoparticles on various substrates would broaden application possibilities.

## Introduction

Lignin, the main aromatic component of lignocellulosic biomass, is a plausible feedstock for various bio-based chemicals and materials. A major driver for these lignin-based materials is the preferred substitution of fossil components. To refine lignin into high-value products, emphasis is placed on developing high-performance, stimuli-responsive materials that exploit its intrinsic functional properties.^1,2^ As interest in using lignin for advanced materials increases, there are many opportunities for innovation across industries, though more work is needed to fully realize its potential.^3^ While generally considered as sustainable alternatives, it is critical to evaluate whether the production processes associated with lignin-based materials are benign or potentially worse compared to their benchmarks.

Close packing of spherical lignin particles^4^ into photonic materials that display structural colors^5^ appears as one of the most promising routes to value-added biobased materials. These photonics share in common features of natural and manmade colloidal crystals such as iridescent opals.^6^ These striking visual appearances makes them appealing for decorative items and anti-counterfeiting applications.^7^

While spherical lignin particles are in the correct size domain, their broad and non-monomodal dispersity presents an obstacle to the spontaneous formation of close-packed phases. Recent advancements include producing monodisperse lignin colloidal spheres that facilitates their assembly into densely packed domains.^5^ Our follow-up work reported particle size classification during centrifugation that drives the packing of the particles into layered rainbow-colored materials.^8^ A significant subsequent advancement was the development of a gradient centrifugation method to fractionate the polydisperse particles before forming the photonic material, providing a simple way to control the observed color.^9^ However, the literature is still lacking a method to produce a single structural color of choice in high yield and using environmentally benign solvents only. Additionally, photonic crystals exhibit angle-dependent colors, which can limit their uses.^10^

As an alternative to colloidal crystals, colloidal glasses exhibit structural colors arising from short-range order. Combined with long-range disorder, the resulting colors appear consistent from any angle.^11^ While photonic glasses can be made into different forms, such as films and 3D-printed objects, they often lean towards softer, pastel colors rather than vibrant shades. Enhancing brightness usually involves increasing long-range order, which can compromise the angle independence.^12–14^ These colors can be easily adjusted by changing the size and arrangement of the particles, making colloidal glasses versatile materials for various applications.^15,16^ For the purpose of structural colors such as biobased glitter or other aesthetically relevant uses it is important to produce the materials in high process mass efficiency and using environmentally benign solvents and processes, all of which are lacking from the current literature.

Here we show that structural colors originating from photonic glass structures can be produced from crude technical lignins using ethanol and water as solvents, giving mass yields in the 48–72% range. We further show that the color of the photonic glass can be tailored through a facile control of water addition rate during the lignin particle preparation. Structural colors including different shades of yellow, blue, and green were obtained in a controllable manner, contributing to their scalable production.

## Results and discussion

### Ethanol as a solvent for dissolving lignin

**Fig. 1:**
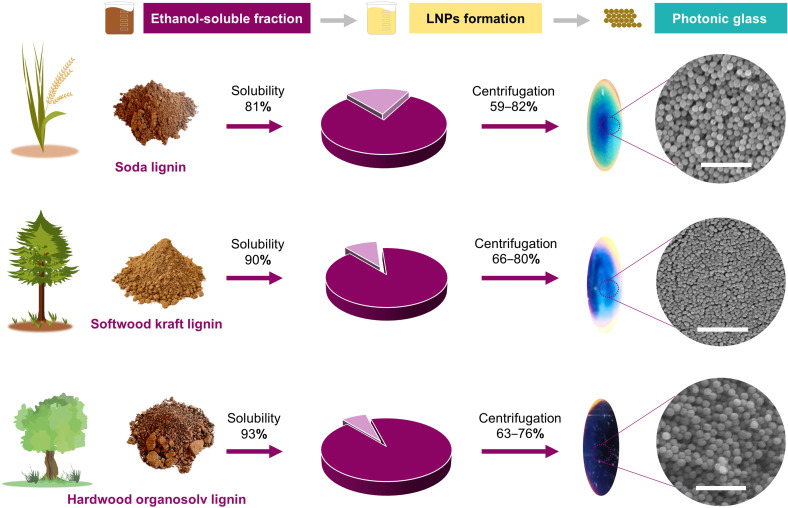
Schematic demonstrating the origin of three different lignins and preparation of the lignin nanoparticle photonic glasses in this work. Lignin powder is extracted using ethanol. The ethanol-soluble fraction was directly used to form lignin nanoparticles (LNP) by adding water. The LNPs were centrifuged to form lignin photonic glasses exhibiting structural color. The percentage values over the arrows indicate the mass yields in the dissolution and centrifugation steps. The scale bars in the SEM micrographs denote 1 μm.

Lignin nanoparticles (LNPs) can be effectively synthesized using solvent shifting. In this process, lignin is dissolved in water-miscible solvents like ethanol or acetone, often with a minor volume fraction of water as a co-solvent. When the volume fraction of organic solvent is reduced the lignin molecules aggregate into spherical nanoparticles. This technique relies on the intermolecular attractive interactions such as pi–pi stacking and other hydrophobic interactions within the lignin structure.^17,18^ Here we used 95% ethanol to directly dissolve 81% of soda lignin, 90% of softwood kraft lignin and 93% of hardwood organosolv lignin relative to their original dry weight (Fig. 1 and Table S1†). Prior to the formation of LNPs, Fourier-transform infrared (FTIR) spectroscopy was further used to compare the chemical structures of soda lignin, softwood kraft lignin, hardwood organosolv lignin and their ethanol-soluble and ethanol-insoluble fractions (Fig. S1–S3†). At 3400 cm^−1^, the broad absorption band corresponding to O–H stretching vibration was observed for all lignin samples. The intensity of this peak is lower for the ethanol-insoluble fraction, notably for hardwood organosolv lignin. Ethanol-soluble lignin showed more prominent O–H stretching peaks than ethanol-insoluble soda lignin, suggesting enrichment of polar lignin molecules in the soluble fraction. The absorption band corresponding to C–H stretching vibration was observed between 2830–2930 cm^−1^. Aromatic skeleton vibrations were observed at the band between 1510–1420 cm^−1^. In the FTIR spectra of hardwood organosolv lignin (Fig. S3†), the band around 830 cm^−1^ corresponds to the C–H out-of-plane in position 2 and 6 of S unit and the band around 1324 cm^−1^ is characteristic for condensed syringyl (S) ring and guaiacyl (G) ring.^19^ Compared to the ethanol-insoluble fraction of the three lignins, the peak between 900–1200 cm^−1^ was markedly reduced in intensity in the case of ethanol-soluble fraction of hardwood organosolv lignin, which could be due to the removal of ethanol-insoluble polysaccharides.

### Particle size control and assembly of LNPs into photonic glasses

**Fig. 2:**
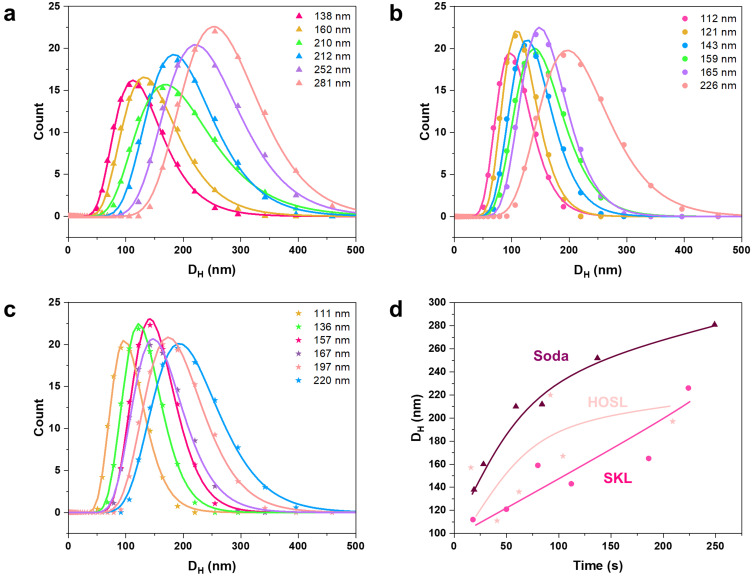
Size distributions and log-normal fits of LNPs from ethanol-soluble fraction of (a) soda lignin, (b) softwood kraft lignin, (c) hardwood organosolv lignin, and (d) hydrodynamic size *vs.* dilution time of lignin nanoparticles formed from the three different lignins (continuous curves are shown for the guide of eye only).

Lignin nanoparticles were prepared through a solvent-shifting method, involving solubilizing lignin in an organic solvent, here ethanol, followed by gradually diluting the solution with water. During this transition, lignin molecules aggregate due to hydrophobic interactions and stacking of their aromatic rings. Other noncovalent forces, such as hydrogen bonding and van der Waals forces, also help stabilize the aggregates. The formation of LNPs depends on the molecular size and solubility of lignin. Larger, less soluble lignin molecules form the hydrophobic core, while smaller, more soluble lignin molecules with hydrophilic groups make up the surface, creating charge repulsion and colloidally stable particles.^17,18^ It is possible to control the particle size by controlling the dilution rate during the formation of LNPs. This control over particle size grants further control over structural coloration of photonic glasses that form from LNPs upon centrifugation. A practical way to control the dilution rate is to vary the time over which three volumes of water relative to the lignin solution in ethanol are mixed. We used these time courses of water addition to label the various lignin nanoparticles and compare their hydrodynamic diameters measured using dynamic light scattering technique. In Fig. 2a–c, the log-normal distribution of hydrodynamic diameters of LNPs formed from soda lignin, softwood kraft lignin and hardwood organosolv lignin from the ethanol-water process at different dilution times and hence different sizes are given, and it is evident that their particle size distributions are broad. It is also apparent that soda lignin formed LNPs with best control over hydrodynamic diameter (Fig. 2d). However, in all cases the LNPs presented colloidally stable particle dispersions that did not sediment under ambient conditions.

**Fig. 3:**
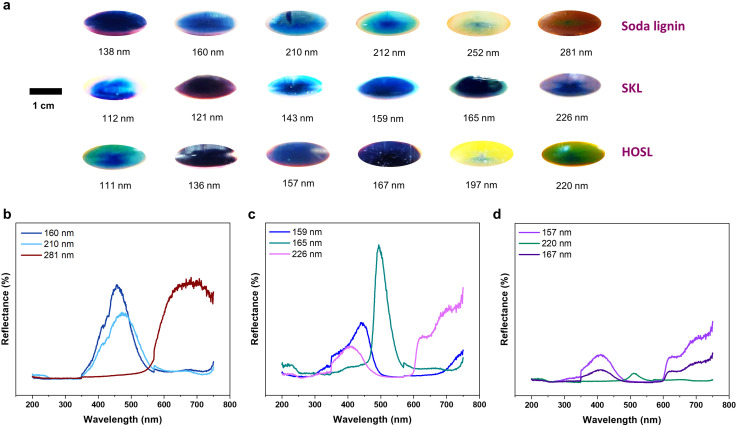
(a) Digital images taken on the polypropylene substrate of the photonic glasses exhibiting structural colors prepared from LNPs with different mean diameters. Reflectance spectra of photonic glasses formed from LNPs from (b) soda lignin, (c) softwood kraft lignin (SKL) and (d) hardwood organosolv lignin (HOSL) having different mean diameters.

To showcase the formation of lignin-based photonic glasses from the dispersions of lignin nanoparticles, we conducted a particle size classification using centrifugation. Stemming from the ability to control the size of soda lignin nanoparticles, the color of the lignin photonic glasses covered a broad visible range (Fig. 3a). These results are in good agreement with the results presented in the PhD thesis of Liu that did not investigate other lignin types.^20^ In contrast, softwood kraft lignin produced structural colors predominantly within the blue-green-purple spectrum, while hardwood organosolv lignin exhibited a broader range, including a distinct yellow color at the longest dilution time during particle formation. Nonetheless, it was possible to produce photonic glass with yellow color for softwood kraft lignin with the tint of green at a particle size of 147 nm–153 nm range (Fig. S5†). The non-iridescent structural colors produced from amorphous material like lignin are mainly blue and violet as can be seen from Fig. 3a. However, colors such as red (620–750 nm) are difficult to obtain, because these occur at wavelengths beyond the range of the photonic band gap of the photonic glass materials; nonetheless, we have obtained brown color with a red tint in the photonic glass derived from soda lignin with a particle size of 249 nm (Fig. S4†).^21^ The vibrant colors observed in Fig. 3a were preserved for up to 8 weeks in polypropylene tubes maintained at 80% relative humidity. Over this period, a slight color dimming was noted. To mitigate this effect, the samples were subsequently stored in a desiccator containing a saturated potassium sulphate solution, which provided a controlled humidity of 94%. Under these conditions, the photonic glass colors appeared unchanged, retaining their original vibrancy as observed immediately after centrifugation (Fig. S7†). In a completely dry state, the photonic glasses tend to fracture into smaller pieces due to the inherent brittleness of lignin. However, upon rewetting, the fragments that retain a uniform layered assembly of lignin nanoparticles again displayed their original color (Fig. S8†).

The mass yield from centrifugation of photonic glasses was correlated with the size of the lignin nanoparticles, which is presented in Table S5.† The results showed that within a type of lignin the yield of photonic glasses from centrifugation increased with the increase in particle size (Fig. S4–S6 and Table S5). Among the three different lignin used, softwood kraft lignin and hardwood organosolv lignin produced higher yields than soda lignin with comparable particle size. The reflectance spectra of LNP photonic glasses agreed with the color observed in the digital images. For soda lignin, the peaks at 456 nm, 473 nm for different shades of blue (160 nm and 210 nm mean particle diameter) respectively and 682 nm for brown corresponding to 281 nm mean particle diameter (Fig. 2a, 3a and 3b). For softwood kraft lignin, the spectra were observed at wavelengths 441 nm (blue, 159 mean diameter), 494 nm (dark green, 165 nm mean particle diameter) and 409 nm (indigo, 226 nm mean particle diameter) as shown in Fig. 2b, 3a and 3c. In the case of hardwood organosolv lignin, the reflectance peak of the photonic glass with observed violet color and formed from particles with a mean diameter of 157 nm showed a peak at 407 nm. When lignin particles with a mean diameter of 220 nm were used, the observed reflectance peak at 510 nm corresponds to the color green, with a tint of yellow, which is observed at the peak around 654 nm. A peak at 408 nm was observed for the photonic glass formed with the particles having a mean diameter of 167 nm resulting in violet color (Fig. 2c, 3a and 3d). Although we decided to work on ethanol as the preferred organic solvent, it is possible to form lignin colloidal photonic glasses with soda lignin, softwood kraft lignin and hardwood organosolv lignin from binary solvent mixtures such as acetone–water (Fig. S9–S11†). Nonetheless, we note that ethanol is a greener solvent compared to acetone.^22^

X-ray diffraction studies confirmed the absence of photonic crystals and demonstrated that the material formed is amorphous with short-range order, classified as photonic glass, as shown in Fig. S21.† It is also worth to note that the digital images of lignin photonic glasses were taken in the wet state. In the dry state, the colors of the photonic glasses are weaker due to increase in coherent scattering reported by Wang *et al.*^5^ Moreover, the photonic glasses formed exhibited non-iridescent structural colors, which are angle-independent. This is again a proof that the structural coloration is not coming from photonic crystals, but from photonic glasses.^15,21^

### Evaluation of photonic glass morphologies

**Fig. 4:**
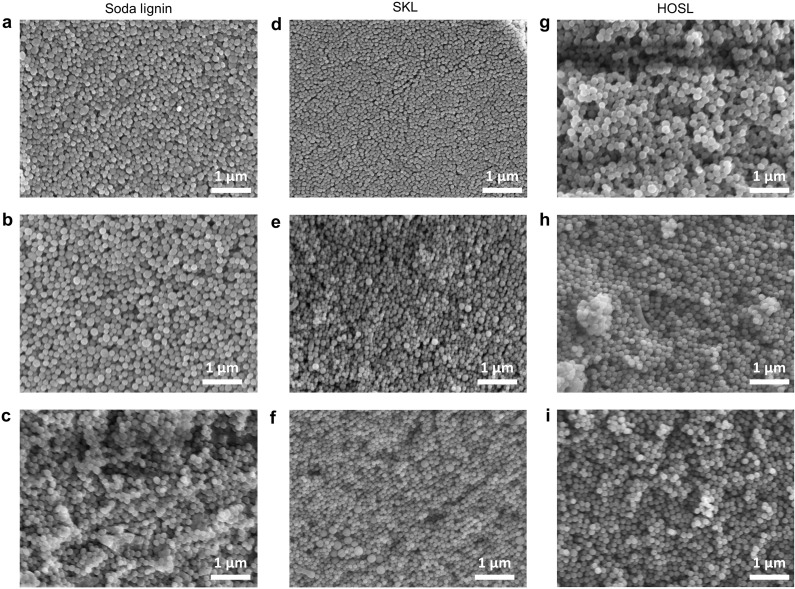
Representative SEM images of lignin photonic glasses from ethanol-water with soda lignin having a mean diameter (a) 160 nm, (b) 212 nm and (c) 281 nm, softwood kraft lignin having mean diameter (d) 112 nm, (e) 121 nm and (f) 226 nm and hardwood organosolv lignin having mean diameter (g) 157 nm, (h) 167 nm and (i) 197 nm respectively.

The nanostructures of lignin-photonic glasses were analyzed using scanning electron microscopy (SEM). When prepared at comparable dilution times (duration over which water was added into the organic solvent containing lignin) apparent differences in particle sizes and appearances were evident among the three lignin sources (Fig. 4). It appeared that the smaller LNPs prepared from soda lignin and softwood kraft lignin were more prone to regular close packing, while the opposite was true for the LNPs from hardwood organosolv lignin. As stated before, it is evident from the microscopic images that photonic glasses are obtained because of the short- range arrangement of nanoparticles with various topological defects; hence, they present an intermediate state between disordered and crystalline structures.^23^ SEM images of more photonic glasses from three different sources of LNPs and at different dilution time are shown in the ESI Fig. S12–S20.† In terms of observed structural coloration, it appears that both the particle size and packing density play a role. It is understood that a fast nucleation of nanoparticles produces more monodisperse nanoparticles, which was also confirmed here with the LNPs.

**Fig. 5:**
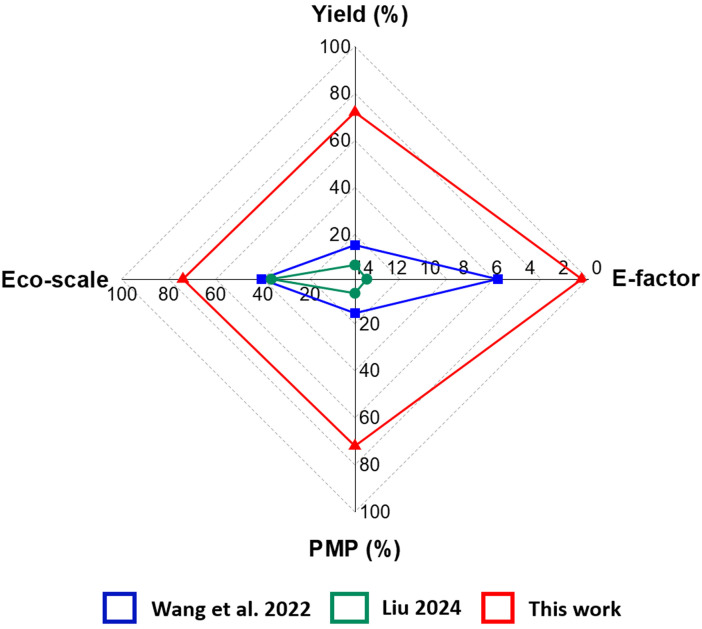
Benchmarking the lignin photonic glass (softwood kraft lignin nanoparticles with a mean diameter of 154 nm) of the present work to the two-step solvent fractionation procedures reported in the literature^20,24^ in terms of yield, environmental factor (E-factor), process mass productivity (PMP) and eco-scale. We note that the previous studies have omitted the centrifugation step from the yield determination, unlike in the present work.

Compared with the previously reported methods for preparing photonic materials from lignin,^8,24^ the methodology reported in this work performed better in the solvent scoring system based on Green Chemistry principles and lower mass of total waste when considering that solvent can be recovered (Fig. 5). Considering the recycling of ethanol, the PMI value obtained in this work is markedly lower compared to the previously reported works as per Fig. 5 and Table S4.† Process Mass Productivity (PMP) is essentially the inverse of Process Mass Intensity (PMI), and is expressed in percentage. In this work, PMP (%) is 67% (soda lignin), 72% (softwood kraft lignin) and 71% (hardwood organosolv lignin). The calculated PMP (%) for work reported in the PhD thesis of Liu (2024) is 7% and Wang *et al.* (2022) is 15.5%. To assess the environmental impact of the materials produced, we calculated the eco-scale scores: 71.5 points for soda lignin, 74 points for softwood kraft lignin, and 73.5 points for hardwood organosolv lignin, which is presented in Table S3.† In comparison, the work of Liu (2024) received 36.5 points, and Wang *et al.* (2022) received 40.75 points on the Eco-Scale. These metrics are evidently in favor of the present work; however, further efforts are required to assess a full-scale production process with respect to the three pillars of sustainability. It is also worth to note, in Fig. 5; the yield for work done by Wang *et al.* (2022) and Liu (2024) was calculated considering no losses in the centrifugation process (a step yield of 100%).^20^ In this work, the overall yield of photonic glass was calculated by multiplying the solubility yield with the actual centrifugation yield. Radar charts comparing different metrices for photonic glasses formation from soda lignin and hardwood organosolv lignin are also presented in the Fig. S22 and S23† respectively.

## Conclusions

We have presented a generalizable method to produce lignin photonic glasses with high-yield using ethanol as a green solvent in the process. The size of lignin nanoparticles can be controlled from 100 nm to 300 nm. The color of the resulting photonic glass can be controlled from violet to brown-red for soda lignin with a yield ranging from 48–67%. Similarly, it is possible to produce lignin photonics with high yield from softwood kraft lignin (60–72%) and hardwood organosolv lignin (58–71%). Due to its simplicity and compliance with green chemistry principles, the method holds potential for scaling up the production of structural colors based on lignin nanoparticles.

## Experimental details

### Materials

Lignin materials (LNPs and structural colors therefrom) in this work were prepared from soda lignin (PROTOBIND 2400, GreenValue LLC, previously characterized by ^31^P NMR spectroscopy),^25^ hardwood organosolv lignin and softwood kraft lignin (BioPiva™100, Finland). Ethanol (95%) from Kiilto, Sweden, and acetone (99.5%) from Honeywell, Germany were used as solvents in the fractionation procedure. Deionized (DI) water was used in all experiments. All chemicals were used as bought unless noted.

### Preparation of lignin nanoparticles (LNPs)

LNPs were prepared by solvent shifting method and the size of LNPs was controlled by varying the total dilution time. Firstly, 256 mg lignin (soda lignin, softwood kraft lignin or hardwood organosolv lignin) was added to 100 mL ethanol and stirred overnight at 600 rpm. The lignin ethanol solution was collected by filtration. Then, to the 50 mL collected lignin ethanol solution 150 mL DI water was added at different speeds *via* a separating funnel, recording the dilution time (soda lignin: 19 s, 28 s, 59 s, 84 s, 137 s and 249 s; softwood kraft lignin: 18 s, 50 s, 80 s, 112 s, 186 s and 224 s; hardwood organosolv lignin: 16 s, 47 s, 62 s, 92 s, 104 s and 209 s) with stirring at 600 rpm. Since the flow rate was not fully controlled, some inaccuracies in the relationship between particle size and dilution time were noted. LNP dispersions were then formed with a controlled size distribution, and named after the corresponding dilution time. Based on DLS analysis the z-average particle diameters of the LNPs were 138 nm, 160 nm, 210 nm, 212 nm, 252 nm and 281 nm (soda lignin), 112 nm, 121 nm, 159 nm, 143 nm, 165 nm and 226 nm (softwood kraft lignin), and 157 nm, 111 nm, 136 nm, 220 nm, 167 nm and 197 nm (hardwood organosolv lignin).

### Preparation of LNP photonic glasses

Lignin photonic glasses were prepared directly from LNPs with broad dispersibility by centrifugation.^8^ Specifically, 200 mL LNP dispersion was added to a 500 mL centrifuge bottle and centrifuged at the force of 3800*g* for 2 h at 20 °C. The supernatant was carefully removed and the pellet was dried at room temperature. The yield of the solid phase was determined gravimetrically.

#### Fourier-transform infrared spectroscopy (FTIR)

A 670-IR spectrometer (Varian, U.S.A, mode: ATR) was used to record the infrared spectra of soda lignin, softwood kraft lignin and hardwood organosolv lignin and their ethanol-soluble and –insoluble fractions within a wavenumber range 400–4000 cm^−1^.

### NMR spectroscopy

^31^P NMR spectra of softwood kraft lignin and hardwood organosolv lignin were recorded using a Bruker Avance Neo 400 MHz spectrometer (Bruker BioSpin GmbH) operating at 400.20 MHz for ^1^H nucleus and at 160.00 MHz for ^31^P nucleus. Sample preparation was done according to our previously reported work and acquisition parameters were: pulse angle 90°, inverse-gated proton decoupled pulse sequence for suppressing NOE, relaxation delay 10 s and 256 scans.^26^ Each sample was recorded in triplicate.

### Dynamic light scattering

A Zetasizer Nano ZS (Malvern, UK) was used to measure the size distribution of LNPs by dynamic light scattering (DLS). 10 μL of LNP dispersion was added to 1 mL DI water. Three replicates of the diluted LNP dispersion samples were measured and the mean values reported.

### UV-Vis spectrophotometry

Agilent Cary 5000 UV-Vis-NIR spectrophotometer was used to measure the reflectance spectra of lignin nanoparticles photonic glasses samples, using praying mantis diffuse reflectance accessory in the wavelength range 200–750 nm.

### X-ray diffraction

Powder X-ray diffraction (XRD) patterns of the lignin photonic materials were obtained using a D8 Discover Diffractometer in reflection mode using Cu Kα radiation (*λ* = 1.5418 Å), 2*θ* ranging from 5° to 120° with an increment of 0.01, without rotating the sample.

### Imaging

A JSM-7000F field emission (Schottky-type FEG) scanning electron microscope was used to image lignin photonic glass samples. An acceleration voltage of 5 kV and a working distance of 10 mm were used. Samples were coated for 120 s with gold using a JFC-1200 fine coater before the SEM study. The gold particles added by sputtering are about 5–15 nm in size. Digital photographs of the lignin photonic glass pellets were acquired using an iPhone 13 Pro digital camera directly after centrifugation of the LNPs.

### Determination of solubility by gravimetric method

A quantity of 256 mg of lignin (fresh basis) was added into 100 mL of ethanol (95%). The water contents of soda lignin (4%), softwood kraft lignin (32%) and hardwood organosolv lignin (3%) were considered in the calculations. The lignin solutions were stirred overnight at 600 rpm. After filtration, 5 mL of each solution were taken to measure the lignin content after dissolution in ethanol. For that, the solutions were dried in the oven at 50 °C. The concentration of lignin per mL was calculated first and then determined the yield in 100 mL which is the total volume. Yield was determined by dividing the dried solid weight by 245.76 mg, 174.08 mg and 248.32 mg for soda lignin, softwood kraft lignin and hardwood organosolv lignin respectively. The solubility was determined to be 81%, 90% and 93% for soda lignin, softwood kraft lignin and hardwood organosolv lignin respectively.

### Determination of photonic glass yield by gravimetric method

After two hours of centrifugation for the preparation of photonic glasses, each supernatant was collected, out of which 10 ml of supernatant is taken in an aluminum pan and kept in oven at 100 °C for 36 h to make sure that only solid residues are left and calculated the concentration of lignin. By knowing the solubility of each type of lignin from the abovementioned procedure, mass of the pellet was calculated by subtracting the lignin present in supernatant from the amount of soluble lignin and thereby calculating the centrifugation yield by dividing the mass of photonic glass pellet by the mass of total soluble lignin.

The overall yield of photonic glass was then calculated by multiplying the solubility yield with the centrifugation yield.

## Author contributions

U.T.V. and M.H.S conceptualized the study. U.T.V., A.J.H., and T.S.P. performed the experiments. U.T.V., A.J.H., T.S.P. and M.H.S. interpreted the data. U.T.V. wrote the first draft. All authors contributed to revising and writing the final version of the manuscript.

## Data availability

Data for this article, including all source data are available at Zenodo at https://doi.org/10.5281/zenodo.14138758.

## Conflicts of interest

There are no conflicts to declare.

## Supplementary Material

GC-027-D4GC05797J-s001
