# Supplementary material for: High-yield production of lignin nanoparticle photonic glasses
Source: Green Chem. 2025 Jan 17;27(7):2130–7. doi: 10.1039/d4gc05797j (PMC11758760; doi:10.1039/d4gc05797j)
Supplement: GC-027-D4GC05797J-s001 [file GC-027-D4GC05797J-s001.pdf]

Supplementary Information (SI) for Green Chemistry.
This journal is © The Royal Society of Chemistry 2025

Supplementary Information

High-yield production of lignin nanoparticle photonic glasses

Unnimaya Thalakkale Veettil, [a] Alberto J. Huertas-Alonso, [a*] Tomás S. Plivelic [b] and Mika H. Sipponen [*a,c]

[a]*Department of Materials and Environmental Chemistry, Stockholm University, SE-106 91 Stockholm, Sweden*

[b]*MAX IV Laboratory, Lund University, Lund, Sweden*

[c]*Wallenberg Wood Science Center, Department of Materials and Environmental Chemistry, Stockholm University, SE-10691 Stockholm, Sweden*

[*]*Corresponding author: mika.sipponen@mmk.su.se; albertojose.huertasalonso@mmk.su.se*

Methods

Calculations of green chemistry metrics

Environmental-factor (E-factor): Water is usually excluded from this calculation. Since ethanol can be recovered easily, has been also excluded.

$$E - factor = \frac{Mass\ of\ waste\ generated\ (kg)}{Mass\ of\ desired\ product\ (kg)} = \frac{Mass\ of\ non{-}soluble\ lignin}{Mass\ of\ soluble\ lignin} \quad (1)$$

For E-factor metric, the optimal value is zero.

Process mass intensity (PMI): PMI is described as follows:

$$PMI = \frac{Total\ mass\ used\ in\ the\ process\ (kg)}{Mass\ of\ desired\ product\ (kg)} = E - factor + 1$$

For PMI metric, the optimal value is 1. (2)

Process mass productivity (PMP) is derived from PMI:

$$PMP\ (\%) = \frac{1}{PMI} X\ 100$$

For PMP metric, the optimal value is 100%. (3)

Table S1. Solubility of lignins in ethanol.

**Lignin sample**	**1**	**2**	**3**	**Average**
Soda Lignin	81.61	78.56	84.00	81 ± 3
Softwood Kraft Lignin	86.84	88.15	94.57	90 ± 4
Hardwood Organosolv Lignin	94.46	92.36	92.04	93 ± 1

Table S2. ^{31}P NMR characterisation of lignin.

Lignin sample	Aliphatic -OH (mmol/g)	Phenolic -OH (mmol/g)	Sum aliphatic and phenolic -OH (mmol/g)	-COOH (mmol/g)
Soda*	1.34 ± 0.02	2.71 ± 0.07	4.05 ± 0.10	0.59 ± 0.01
Softwood Kraft Lignin	1.89 ± 0.02	4.01 ± 0.07	5.90 ± 0.09	0.49 ± 0.01
Hardwood Organosolv Lignin	3.49 ± 0.14	2.21 ± 0.03	5.70 ± 0.13	0.13 ± 0.01

* Data extracted from *ACS Appl. Mater. Interfaces* **2022**, *14*, 12693–12702[1]

Table S3. Eco-scale calculation for corresponding to the formation of lignin nanoparticle photonic glasses from soda lignin, softwood Kraft lignin and hardwood organosolv lignin.

**Calculation of penalty points (This work)** *			
**Parameter**	**Penalty points**		
	**Soda lignin**	**SKL**	**HOSL**
**Reaction yield**	16.5 (67% yield)	14 (72 % yield)	14.5 (71 % yield)
**Price of reaction components (to obtain 10 mmol final product)** Lignin & ethanol	0	0	0
**Safety**. Ethanol (T, F)	10	10	10
**Technical setup** Instruments for controlled addition of chemicals	1	1	1
**Temperature/time** Room temperature, < 24 h	1	1	1
**Workup and purification** Removal of solvent with bp < 150°C	0	0	0
**Total penalty points**	**28.5**	**26**	**26.5**

*Calculations are done based on the highest particle size as per Table S5.

Table S4. Summary of Green Chemistry Performance Metrics calculated in this work corresponding to the formation of lignin nanoparticle photonic glasses from soda lignin, softwood Kraft lignin and hardwood organosolv lignin and their comparison with related literature.

	**This work * (soda lignin)**	**This work * (SKL)**	**This work * (HOSL)**	**Liu** 2024**	**Wang *et al.* 2022**	**Wang *et al.* 2023**
**E-factor**	0.49	0.39	0.41	13.29	5.45	5.45
**PMI**	1.49	1.39	1.41	14.29	6.45	6.45
**PMP**	67	72	71	7	15.5	15.5
**Eco-scale**	71.5	74	73.5	36.5	40.75	40.75

*Calculations are done based on the highest particle size as per Table S5.

** Extracted from J. Liu, Doctoral dissertation, Stockholm University, Stockholm, 2024.

Table S5. Correlation between overall yield of photonic glasses with the size of the nanoparticles from which they are formed.

**Lignin sample**	**Particle Size (nm)**	**Overall Yield* (%)**
Soda Lignin	130.3	48
	142.2	53
	249.2	67
Softwood Kraft Lignin	105.3	60
	147.5	64
	153.5	72
Hardwood Organosolv Lignin	147.4	58
	163.3	70
	164.2	71

*Overall yield = Solubility yield * Centrifugation yield

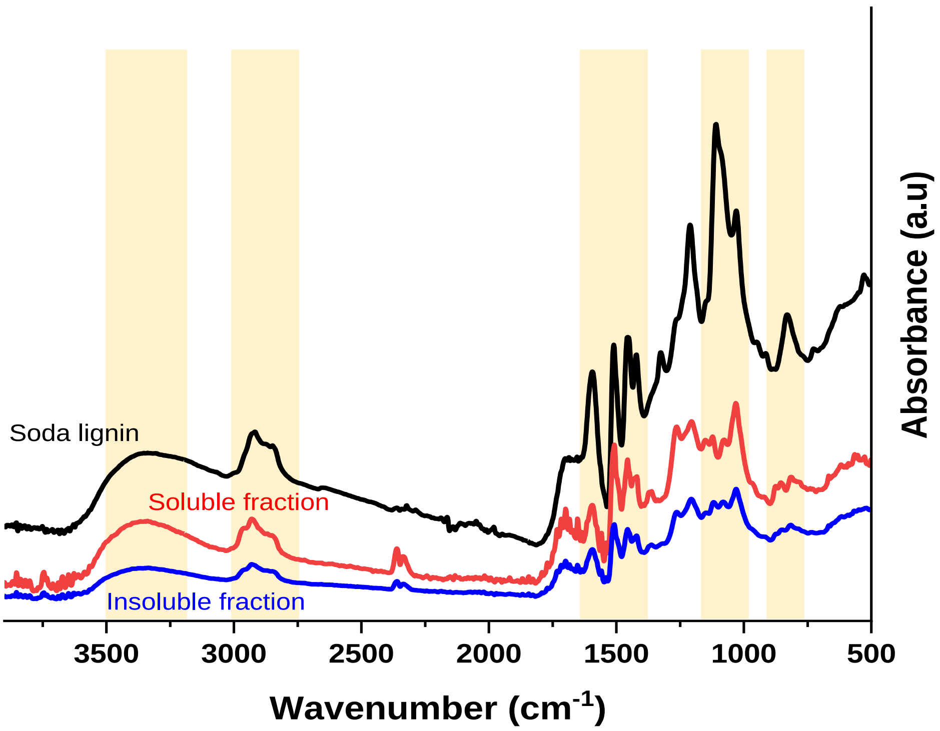

Figure S1. FTIR spectra of soda lignin and its ethanol-soluble and ethanol-insoluble fractions.

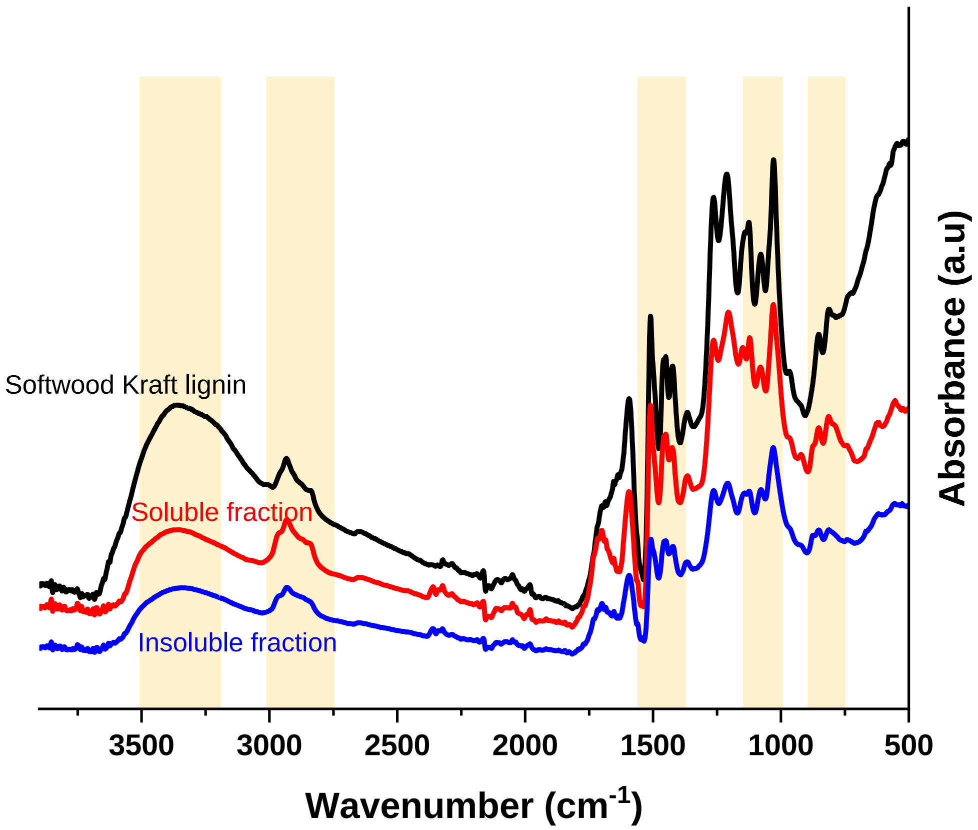

Figure S2. FTIR spectra of softwood Kraft lignin samples lignin and its ethanol-soluble and ethanol-insoluble fractions.

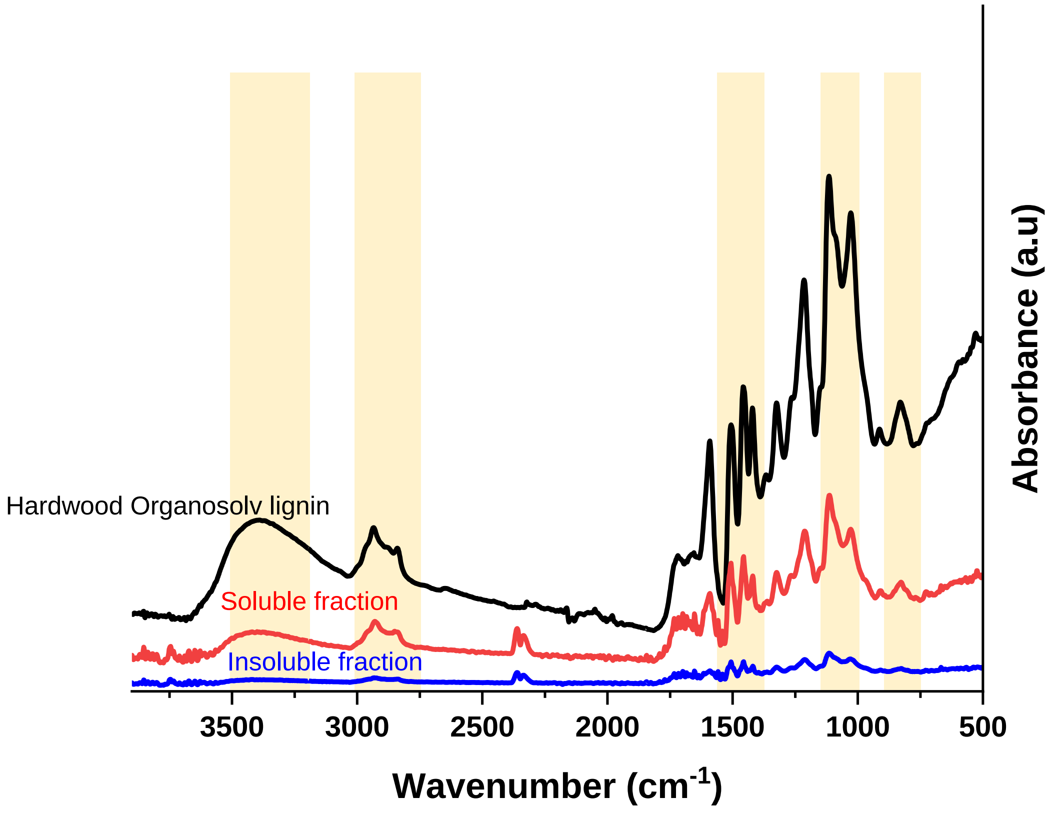

Figure S3. FTIR spectra of hardwood organosolv lignin and its ethanol-soluble and ethanol-insoluble fractions.

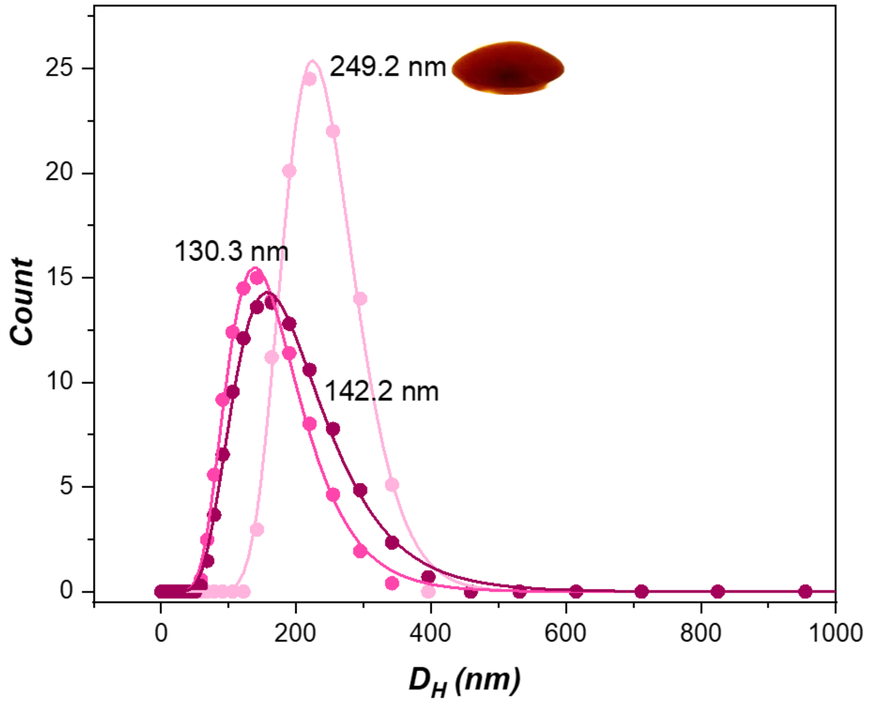

Figure S4. Size distribution of lignin nanoparticles formed from soda lignin, which used to make photonic glasses to calculate the yield. Digital image of photonic glass having brown coloration with tint of red is in the inset.

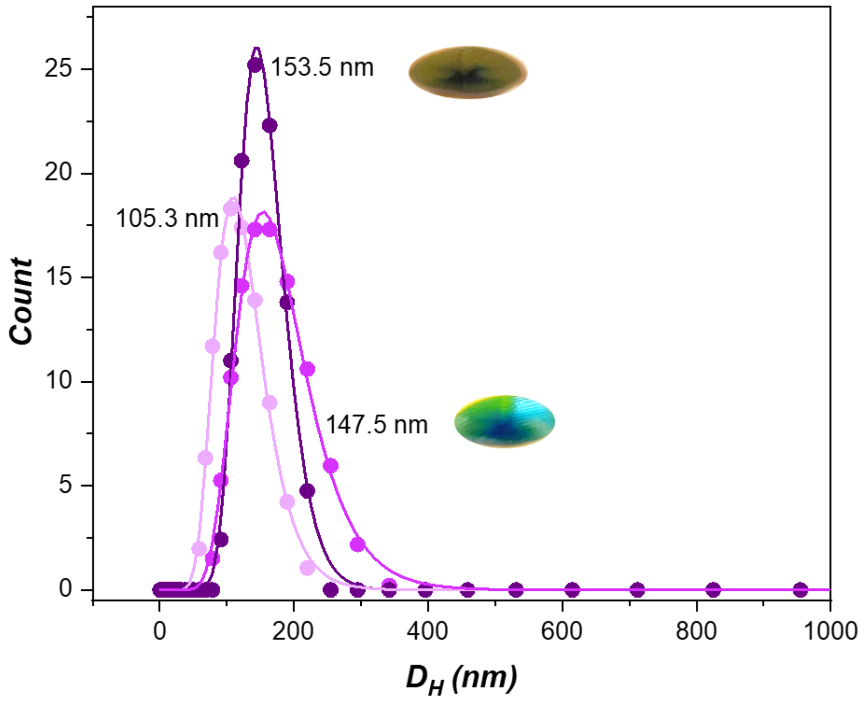

Figure S5. Size distribution of lignin nanoparticles formed from softwood Kraft lignin, which used to make photonic glasses to calculate the yield. Digital image of photonic glasses having yellow coloration with tint of green in the inset.

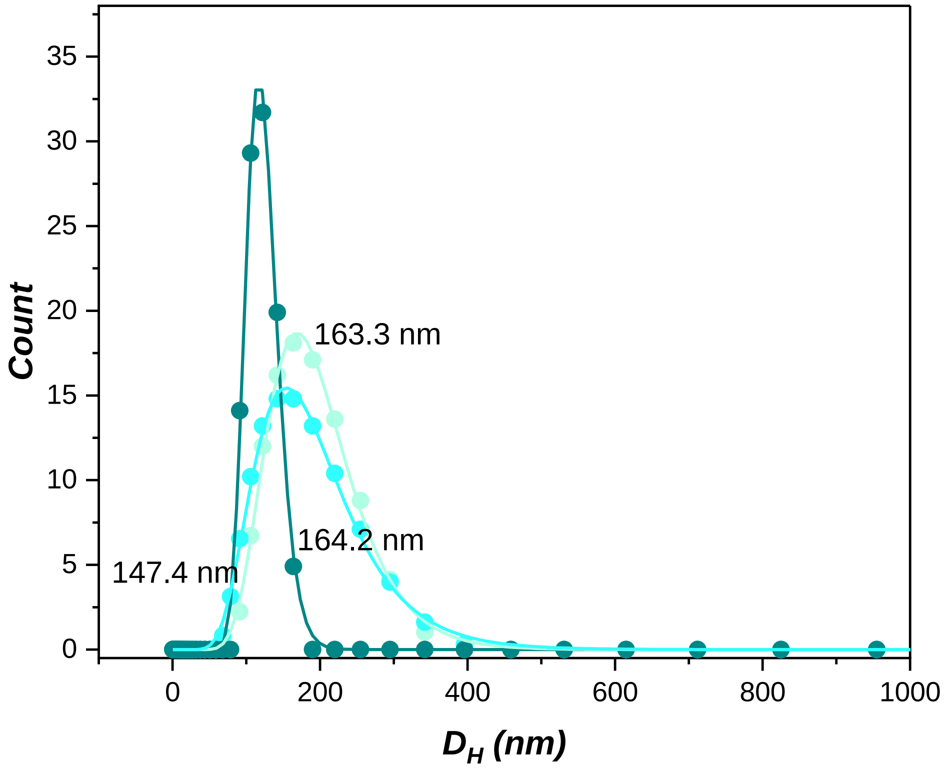

Figure S6. Size distribution of lignin nanoparticles formed from hardwood organosolv lignin, which used to make photonic glasses to calculate the yield.

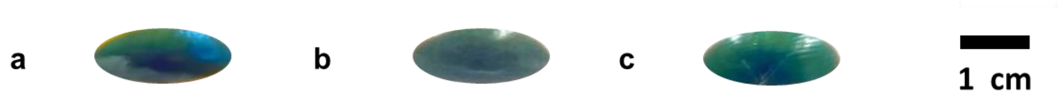

Figure S7. Digital images of lignin nanoparticle photonic glass formed from soda lignin: (a) immediately after preparation, (b) after 8 weeks of storage at 80% relative humidity, and (c) after 24 hours at 94% relative humidity in a desiccator containing a saturated potassium sulphate solution.

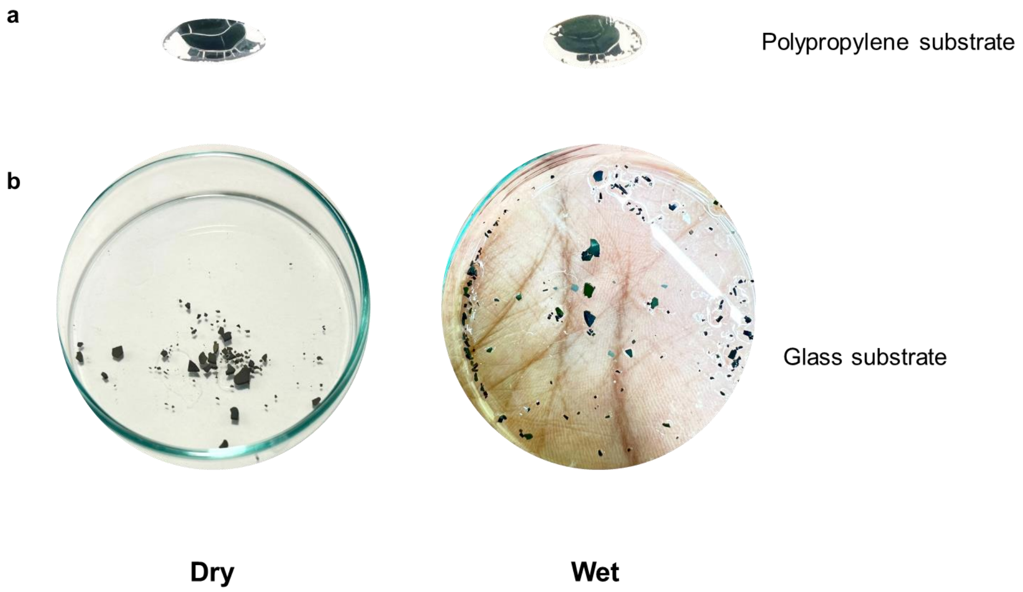

Figure S8. Digital images of lignin nanoparticle photonic glass fragments formed from soda lignin: (a) on a polypropylene substrate, and (b) on a glass substrate. Left side shows the completely dried state of the photonic glasses and right side shows the images after rewetting the photonic glasses.

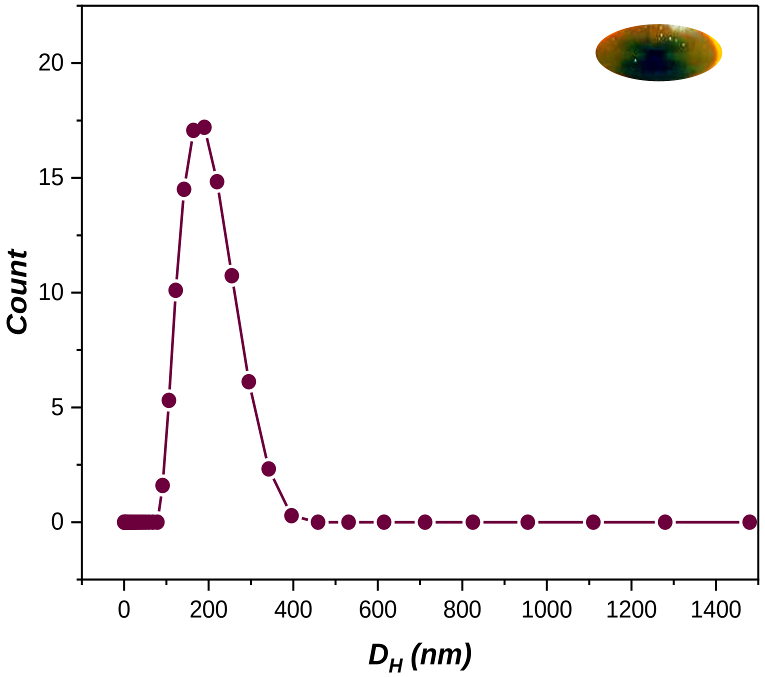

Figure S9. Size distribution of lignin nanoparticles formed from acetone-water using soda lignin with a dilution time of 20s. A representative digital photograph of soda lignin nanoparticle photonic glass in the inset.

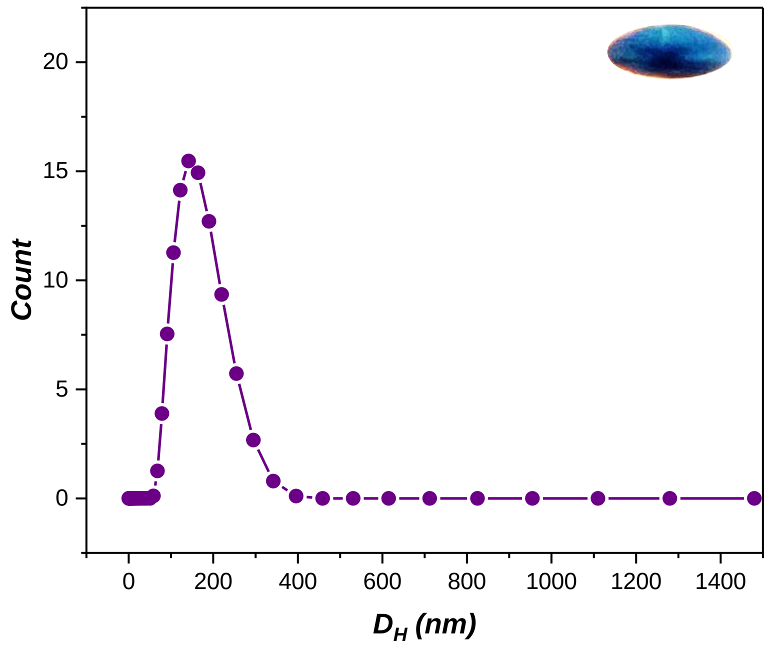

Figure S10. Size distribution of lignin nanoparticles formed from acetone-water using softwood Kraft lignin with a dilution time of 20s. A representative digital photograph of softwood Kraft lignin nanoparticle photonic glass in the inset.

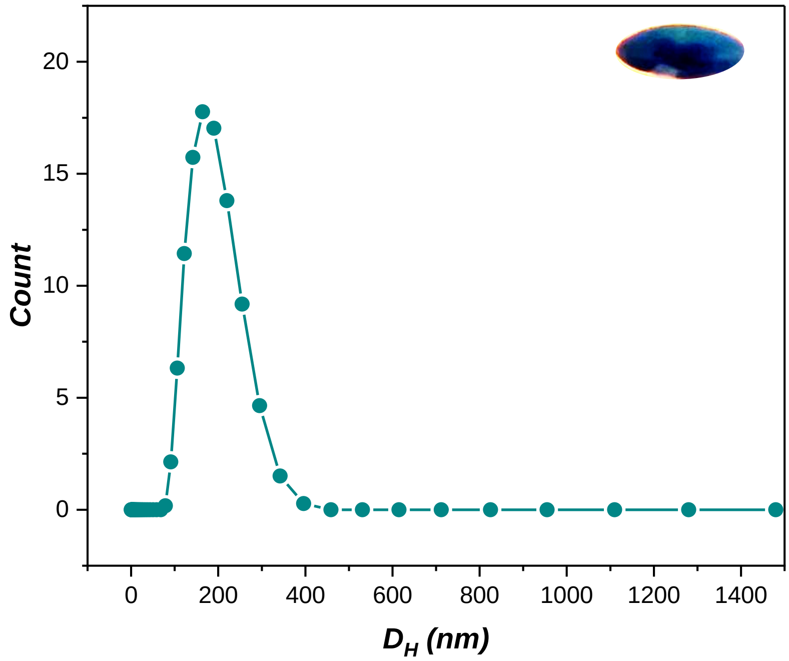

Figure S11. Size distribution of lignin nanoparticles formed from acetone-water using hardwood organosolv lignin with a dilution time of 20s. A representative digital photograph of hardwood organosolv lignin nanoparticle photonic glass in the inset.

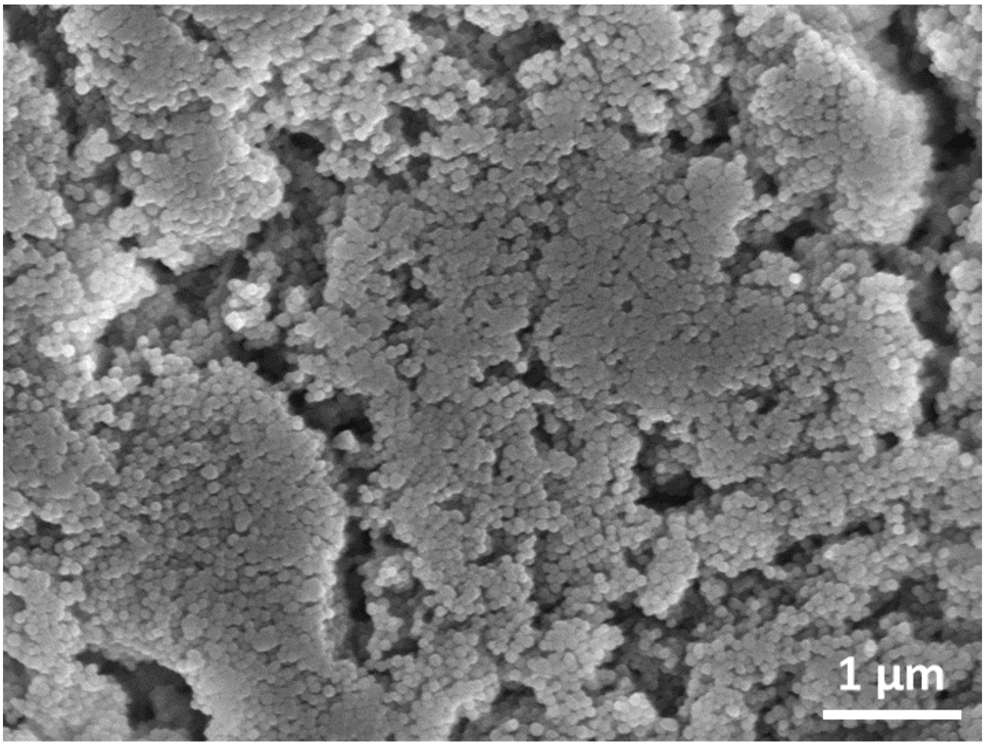

Figure S12. SEM image of photonic glasses formed from soda lignin nanoparticles from ethanol-water solvent shifting process corresponding to a mean diameter 138 nm.

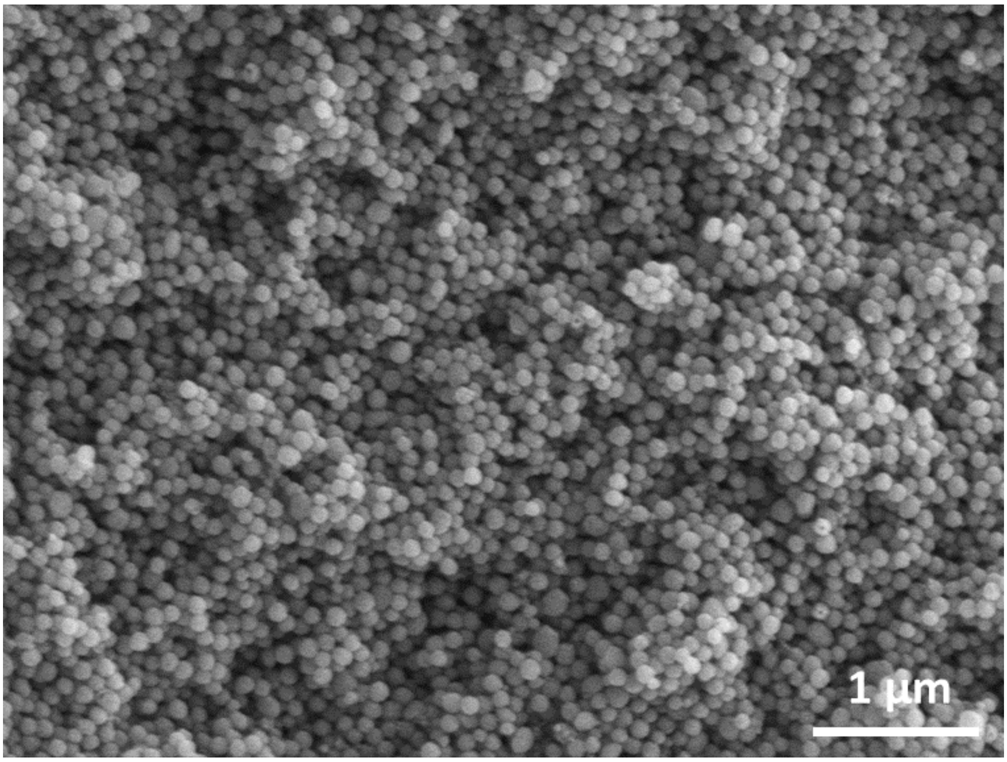

Figure S13. SEM image of photonic glasses formed from soda lignin nanoparticles from ethanol-water solvent shifting process corresponding to a mean diameter 210 nm.

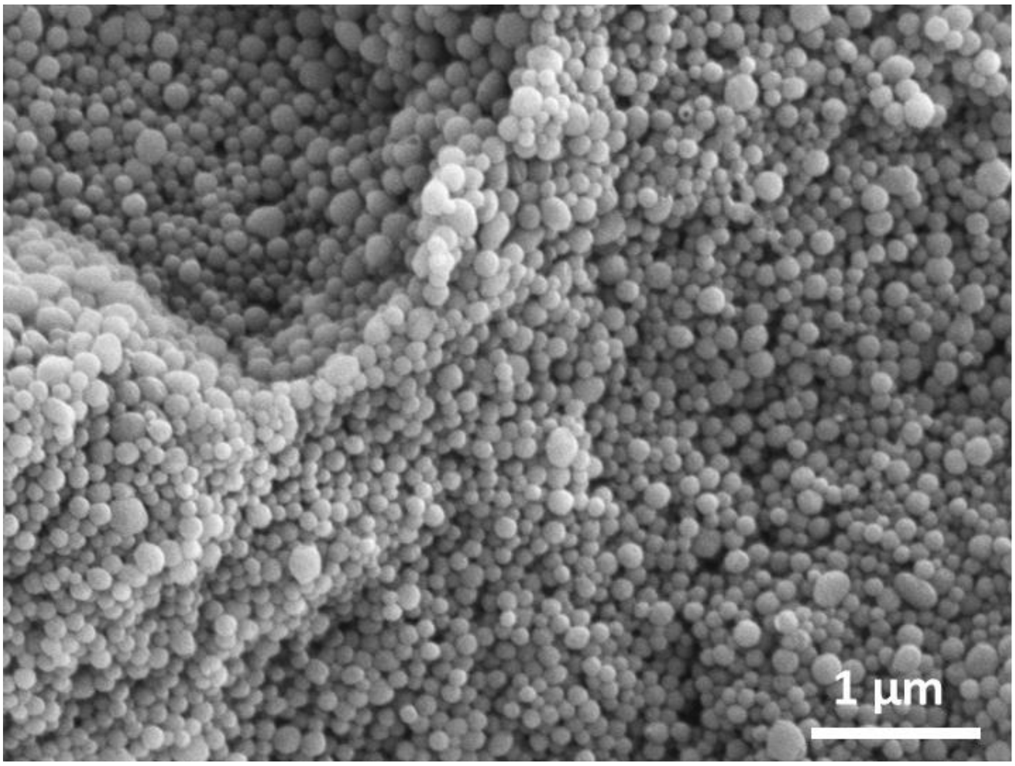

Figure S14. SEM image of photonic glasses formed from soda lignin nanoparticles from ethanol-water solvent shifting process corresponding to a mean diameter 152 nm.

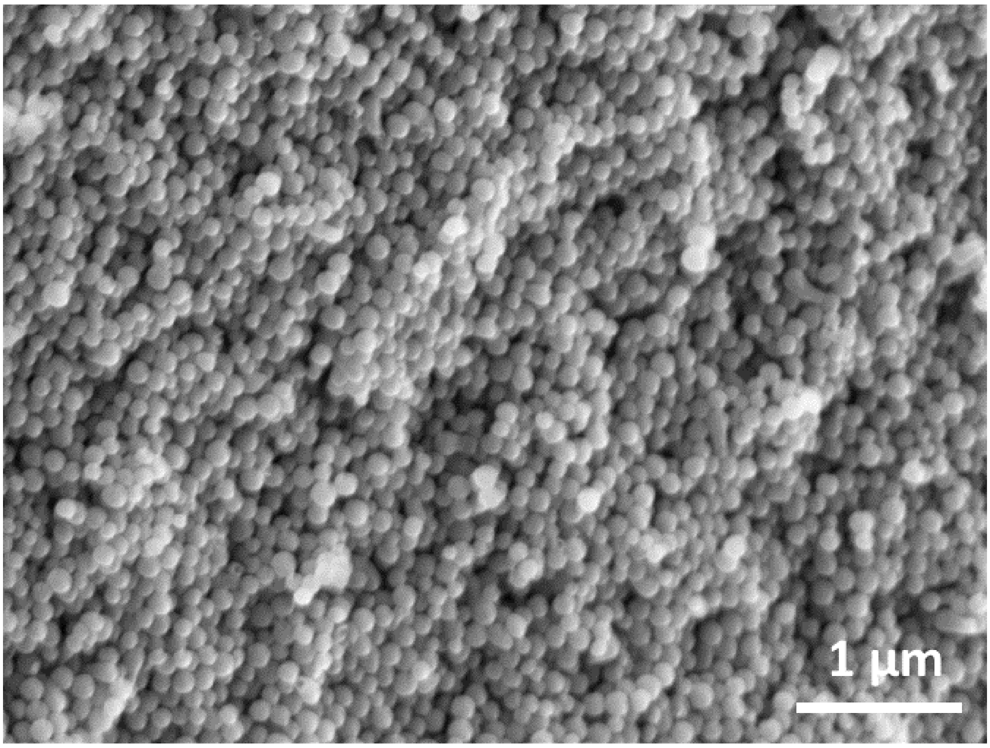

Figure S15. SEM image of photonic glasses formed from softwood Kraft lignin nanoparticles from ethanol-water solvent shifting process corresponding to a mean diameter 159 nm.

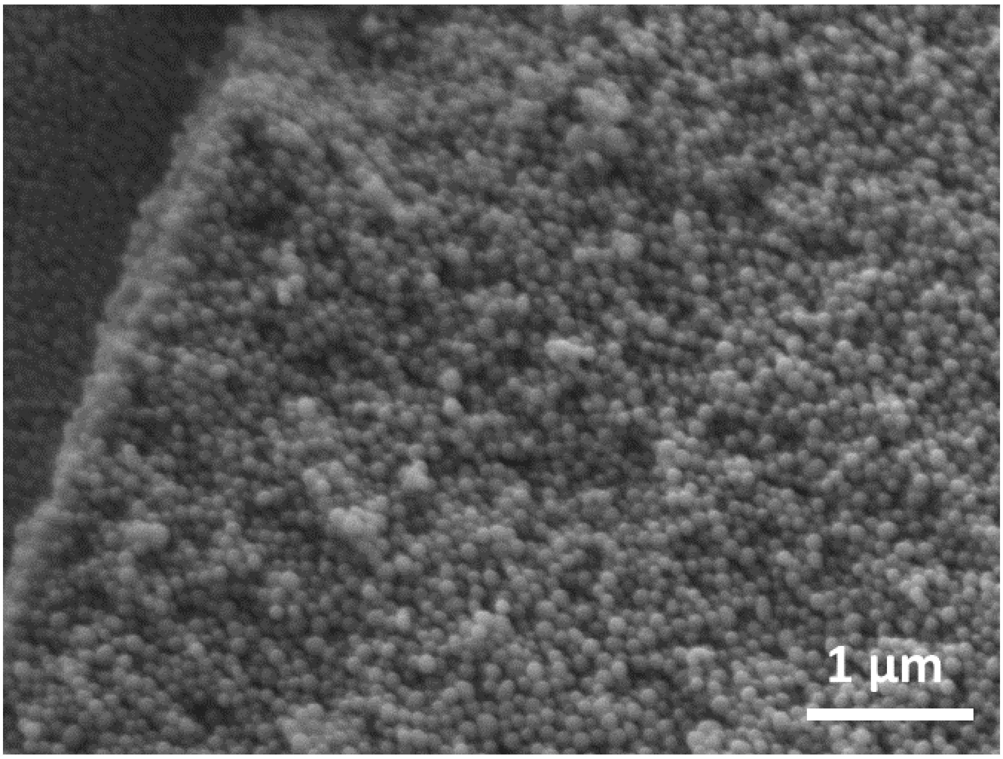

Figure S16. SEM image of photonic glasses formed from softwood Kraft lignin nanoparticles from ethanol-water solvent shifting process corresponding to a mean diameter 143 nm.

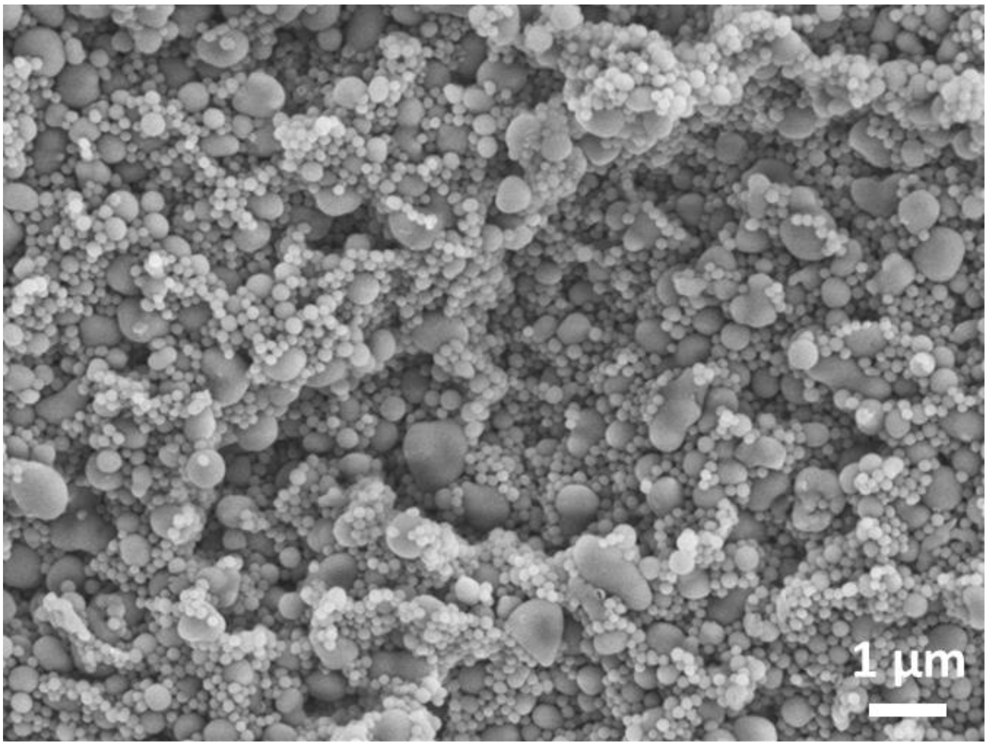

Figure S17. SEM image of photonic glasses formed from softwood Kraft lignin nanoparticles from ethanol-water solvent shifting process corresponding to a mean diameter 165 nm.

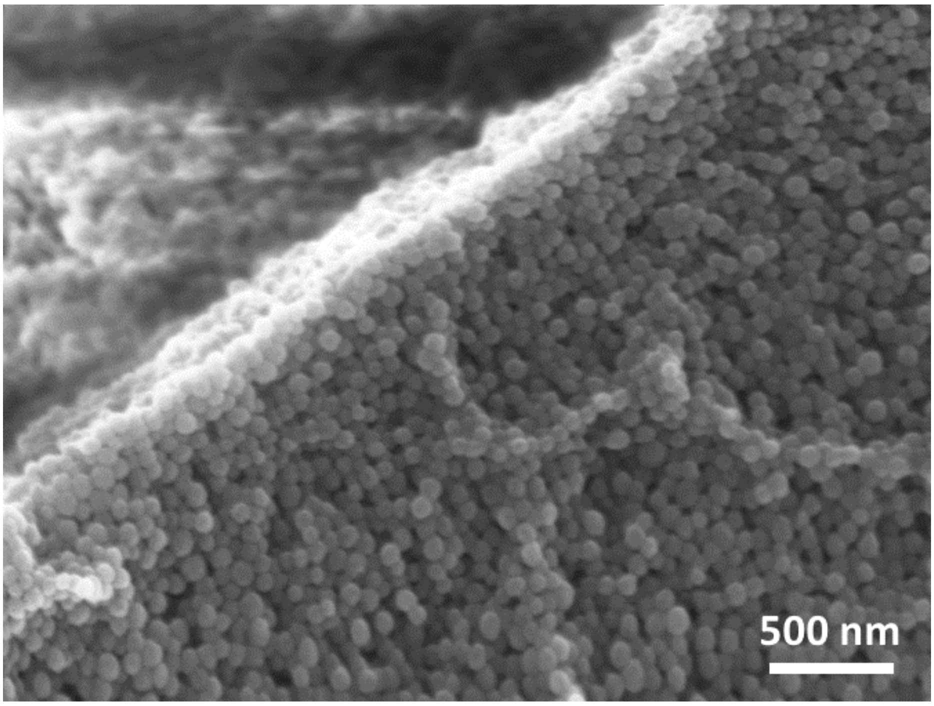

Figure S18. SEM image of photonic glasses formed from hardwood organosolv lignin nanoparticles from ethanol-water solvent shifting process corresponding to a mean diameter 111 nm.

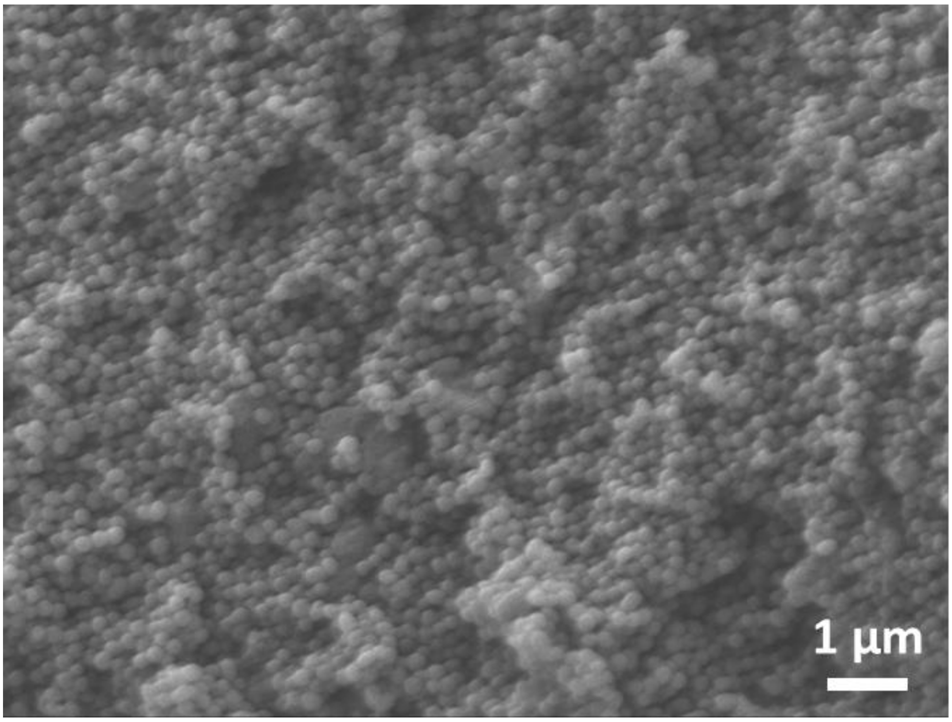

Figure S19. SEM image of photonic glasses formed from hardwood organosolv lignin nanoparticles from ethanol-water solvent shifting process corresponding to a mean diameter 136 nm.

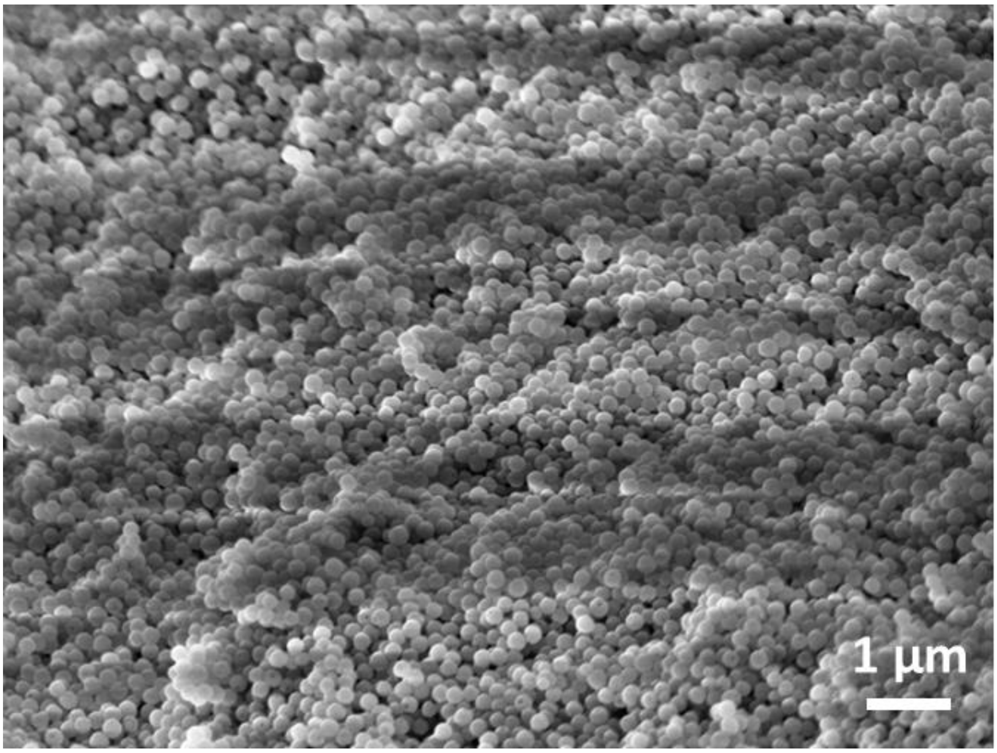

Figure S20. SEM image of photonic glasses formed from hardwood organosolv lignin nanoparticles from ethanol-water solvent shifting process corresponding to a mean diameter 220 nm.

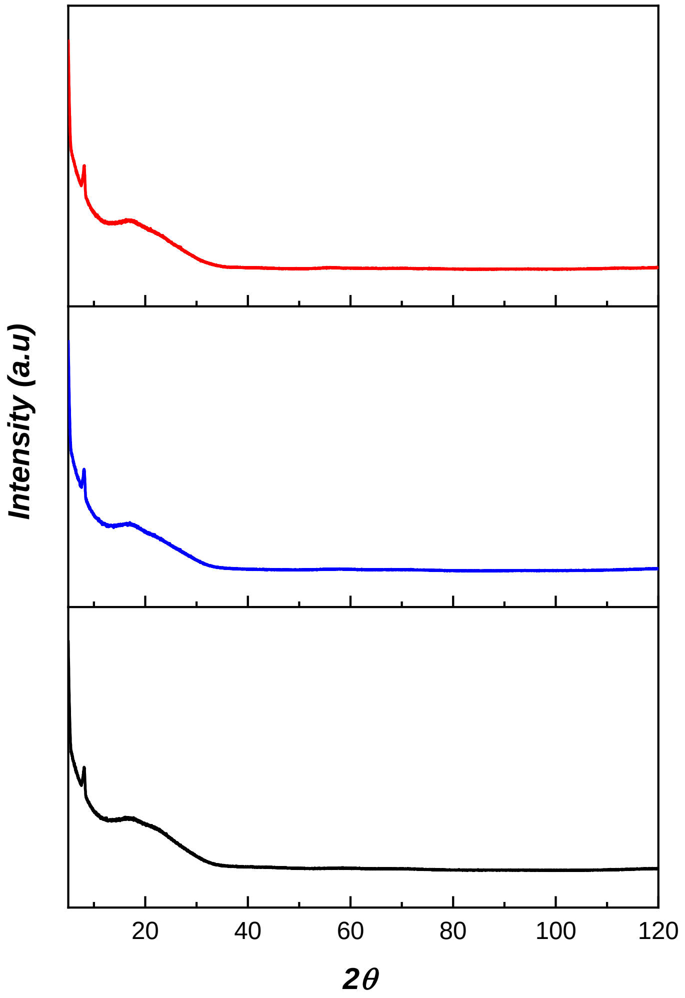

Figure S21. X-ray diffractograms of lignin nanoparticle photonic glasses formed from soda lignin (red), softwood Kraft lignin (blue) and organosolv lignin (black).

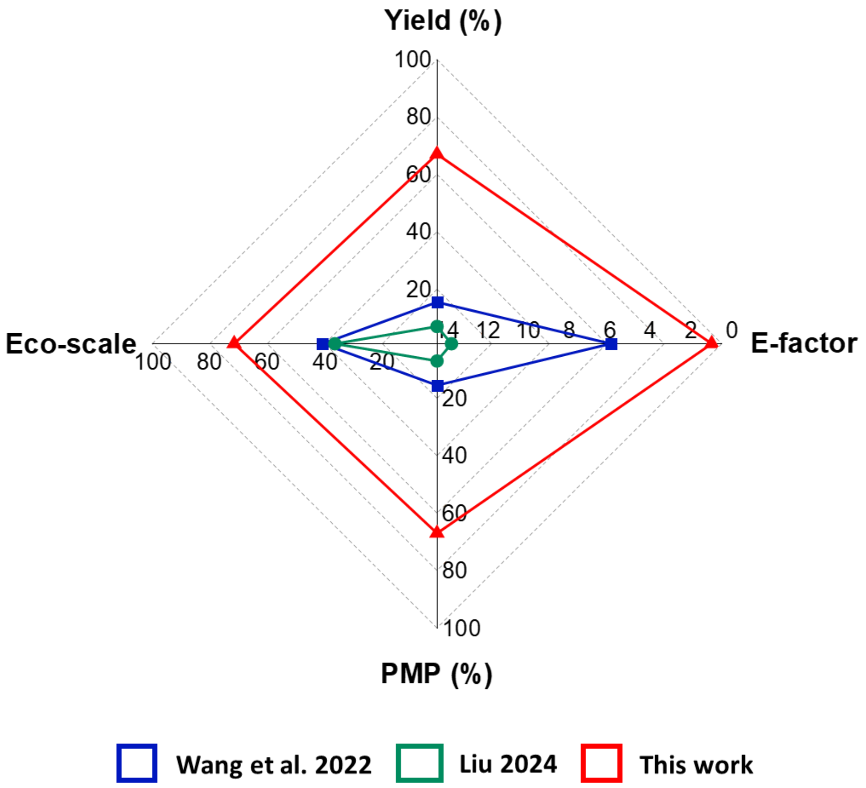

Figure S22. Benchmarking the lignin photonic glass (soda lignin nanoparticles with a mean diameter of 249.2 nm) of the present work to the literature[2–4] in terms of yield, Environmental factor (E-factor), Process Mass Productivity (PMP) and Eco-scale.

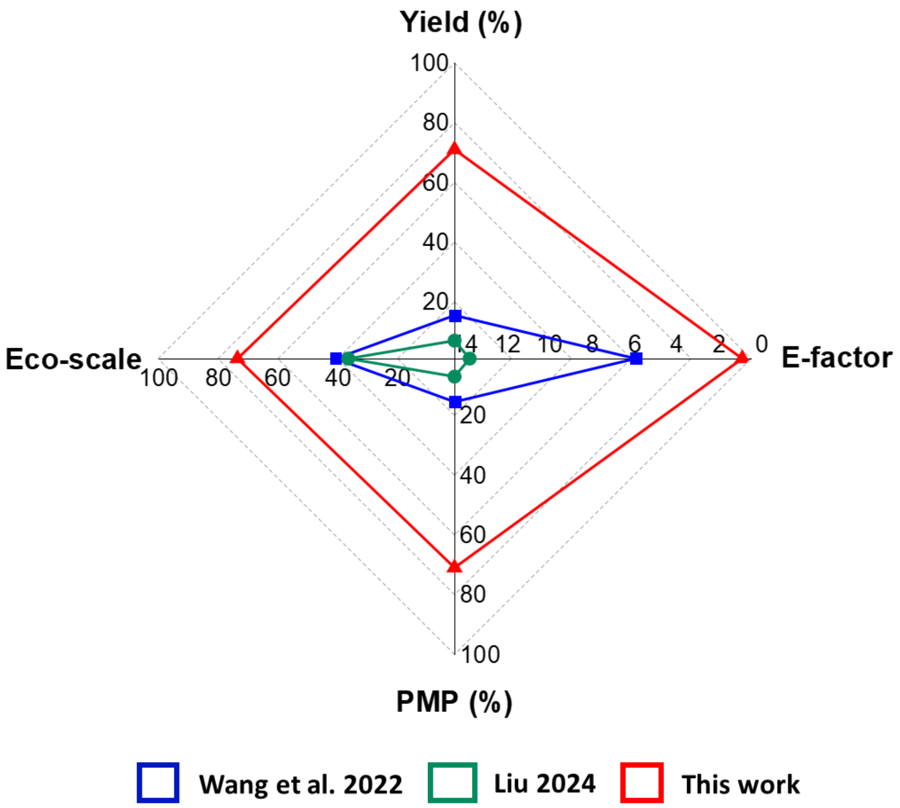

Figure S23. Benchmarking the lignin photonic glass (hardwood organosolv lignin nanoparticles with a mean diameter of 164.2 nm) of the present work to the literature[2–4] in terms of yield, Environmental factor (E-factor), Process Mass Productivity (PMP) and Eco-scale.

References

1. Liu, J., Moreno, A., Chang, J., Morsali, M., Yuan, J., and Sipponen, M.H. (2022). Fully Biobased Photothermal Films and Coatings for Indoor Ultraviolet Radiation and Heat Management. ACS Appl. Mater. Interfaces *14*, 12693–12702. https://doi.org/10.1021/acsami.2c00718.

2. Wang, J., Chen, W., Yang, D., Fang, Z., Liu, W., Xiang, T., and Qiu, X. (2022). Photonic Lignin with Tunable and Stimuli- Responsive Structural Color. https://doi.org/10.1021/acsnano.2c07756.

3. J. Liu, Doctoral dissertation, Stockholm University, Stockholm, 2024.

4. Wang, J., Chen, W., Yang, D., Fang, Z., Liu, W., Xiang, T., and Qiu, X. (2022). Monodispersed Lignin Colloidal Spheres with Tailorable Sizes for Bio-Photonic Materials. Small *2200671*, 1–11. https://doi.org/10.1002/smll.202200671.
